# Detection of *Bacillus anthracis* and B*acillus anthracis*-like spores in soil from state of Rio de Janeiro, Brazil

**DOI:** 10.1590/0074-02760200370

**Published:** 2020-11-06

**Authors:** Jacqueline RS Salgado, Leon Rabinovitch, Maria de Fátima dos S Gomes, Regina Celia da SB Allil, Marcelo Martins Werneck, Rafael B Rodrigues, Renata C Picão, Fernanda Baptista de Oliveira Luiz, Adriana M Vivoni

**Affiliations:** 1 Exército Brasileiro, Instituto de Defesa Química, Biológica, Radiológica e Nuclear, Laboratório de Defesa Biológica, Rio de Janeiro, RJ, Brasil; 2 Fundação Oswaldo Cruz-Fiocruz, Instituto Oswaldo Cruz, Laboratório de Fisiologia Bacteriana/Laboratório de Referência Nacional para Carbúnculo, Rio de Janeiro, RJ, Brasil; 3 Universidade Federal do Rio de Janeiro, Instituto Alberto Luiz Coimbra de Pós-Graduação e Pesquisa de Engenharia, Laboratório de Instrumentação e Fotônica, Rio de Janeiro, RJ, Brasil; 4 Universidade Federal do Rio de Janeiro, Instituto de Microbiologia Paulo de Góes, Rio de Janeiro, RJ, Brasil

**Keywords:** spores, Bacillus anthracis, *Bacillus anthracis*-like, anthrax and soil

## Abstract

**BACKGROUND:**

*Bacillus anthracis* is the aetiologic agent of anthrax, a re-emerging, septicaemic, haemorrhagic and lethal disease that affects humans, domestic ruminants and wildlife. Plasmids pXO1 and pXO2 are attributes that confer pathogenicity to *B. anthracis* strains. This bacterium was used as biological weapon in the World Wars and in the biological attack in the United States of America at 2001. *B. anthracis* is classified as a Tier 1 bioterrorism agent by the Centers for Diseases Control and Prevention. Anthrax is recognised as a re-emerging disease. Several studies concerning the dynamics of *B. anthracis* cycle in soil revealed that nonpathogenic *B. anthracis* strains due to lack of pXO2 plasmid are commonly found in some types of soil.

**OBJECTIVES:**

This study aimed isolation and identification of *B. anthracis* spores in soil samples of the state of Rio de Janeiro, Brazil.

**METHODS:**

Phenotypic and genotypic approaches were used to identify isolates including MALDI-TOF/MS, motility test, susceptibility to gamma phage and penicillin, survey for *pag* and *cap* genes as surrogates of pXO1 and pXO2 plasmids, respectively, and sequencing of 16SrRNA-encoding gene. Physicochemical analysis of the soil samples were carried out to describe soil characteristics.

**FINDINGS:**

We observed the presence of one *B. anthracis* pXO1+ and pXO2- isolated from clay loam soil; one *B. anthracis*-like strain pXO1+ and pXO2-isolated from loamy sand; and 10 *Bacillus* spp. strains sensitive to phage-gamma that need better characterisation to define which their species were recovered from loamy sand.

**MAIN CONCLUSIONS:**

This work showed promising results and it was the first study to report results from an active surveillance for *B. anthracis* in Brazil.

*Bacillus anthracis* is a non-motile, non-haemolytic, aerobic Gram-positive endospore-forming rod. It belongs to the *B. cereus* group, which comprises at least eight closely related species: *B. anthracis*, *B. cereus*, *B. thuringiensis*, *B. mycoides*, *B. pseudomycoides*, *B. weihenstephanensis*, *B. cytotoxicus* and *B. Toyonensis.* ^1^ *B. anthracis* pathogenicity is mainly due to its ability to produce toxins and capsule, which are encoded by pXO1 and pXO2 plasmids, respectively. It is the aetiologic agent of anthrax, a zoonotic, septic, haemorrhagic and lethal disease that affects mostly domestic and wild ruminants.^2^ *B. anthracis* is ubiquitous in nature and the spores are resistant to drying, radiation and disinfectants. 

*B. anthracis* was used in bioweapon programs of many countries, such as Germany during World War I, Japan in World War II, the former Union of Soviet Socialist Republics (USSR) (1928-1992), United States of America (USA) (1941-1969), Iraq (1970-1991) and others. In addition, it was used in bioterrorist attacks perpetrated by Aum Shinrikyo (1995, Japan, no fatalities) and the Amerithrax (2001, USA, five people killed and 22 infected). It is classified by the Centers for Disease Control and Prevention (CDC) as a Tier 1 biological agent.^3^ ^,^ ^4^

It can be isolated from environmental sources such as soil and water, and from food products. *B. anthracis* spores can remain viable for years in soils with pH between 6 and 8.5, especially at the deeper layers. Changes such as plowing or drainage, however, carry them to the surface. Outbreaks of anthrax are frequent in tropical and subtropical countries with high annual rainfalls and are common after major changes in weather, such as heavy rains after a long period of drought, or a dry summer after heavy rains, always in temperatures above 15°C. Soil conditions such as pH, salts, organic matter, temperature, humidity and microbial burden vary according climatic seasons.^5^ Regardless of how *B. anthracis* spores reach the ground, it is generally accepted that some soils are more likely to harbour spores than others. *B. anthracis* is most frequently found in clayey soils rich in organic matter and Ca^2+^, with pH above 6.0 and temperatures above 15.5°C.^6^ ^,^ ^7^

According to Schild et al.,^8^ the disease occurs all over South America where Brazil share borders (total length of 16,885.7 km) with 10 out of 12 countries. From 2006 until July 2019, Argentina (borders with Rio Grande do Sul, Santa Catarina e Paraná states) reported 144 anthrax outbreaks, Uruguay (borders with Rio Grande do Sul state) 63 outbreaks, Paraguay (borders with Paraná e Mato Grosso do Sul states) 54 outbreaks, Peru (borders with Acre e Amazonas states)18 outbreaks, Bolivia (borders with Mato Grosso e Rondônia states) 38 outbreaks and Colombia (border with Amazonas state) 11 outbreaks.^9^ Brazil’s vast border extension can make it vulnerable to the clandestine entry of contaminated animals.

In Brazil, anthrax is on the list of diseases that requires animal health protection measures since 1934, requiring the sacrifice of affected animals and mandatory notification. According to the Ministry of Agriculture, Livestock and Supply, the last reported animal case occurred in 2016, in the state of Rio de Janeiro.^10^ Regarding to human disease, nine cases of cutaneous anthrax were reported, between 1930-1932, all due to contact with contaminated animals.^11^

Since 1928, anthrax in cattle and goats mostly occurs in three distinct areas of Brazil: south and west region of Rio Grande do Sul, where some places were called “cursed fields”; Paraiba river valley in São Paulo and Minas Gerais states; and in the northeast region. Among 72 outbreaks of sudden death in cattle that occurred between 2000 and 2014 in Rio Grande do Sul, seven were identified as caused by *B. anthracis*.^12^ Ten outbreaks of anthrax were confirmed from January 1978 to March 2006 in Brazil occurred in cattle, most non-vaccinated, in the southeastern and southern region of Rio Grande do Sul, in municipalities on the Uruguay border. Lack of vaccination may have been an adjuvant for the occurrence of the disease after exposure to a primary source, such as soil from an old anthrax grave.^8^

Several studies on the dynamics of *B. anthracis* cycle in soil have been carried out.^7^ ^,^ ^13^ However, there is no data available about the occurrence of *B. anthracis* in soil in Brazil and very little information about Brazilian strains. The aim of this research was to survey *B. anthracis* in soil samples from Brazil, correlated or not with anthrax cases.

## MATERIALS AND METHODS

*Soil samples -* Soil samples were collected at two different geographical sites in Rio de Janeiro state: in Barra de Guaratiba [three soil samples, two from Fazenda Modelo and one from Army Technology Center (CTEx)], sites not correlated with anthrax cases and in Barra do Piraí, location of the last case of anthrax reported in Brazil, in 2016, a cattle burrier site. Table I shows geographical coordinates of sampling.

**TABLE I:** Geographic coordinates of soil sampling

Code^*a*^	Location	Geographic coordinates (latitude/longitude)^*b*^
BP1	Barra do Piraí	22.41433/43.56128
BP2	Barra do Piraí	22.41429/43.56114
BP3	Barra do Piraí	22.41440/43.56129
CT	Barra de Guaratiba	23.030097/43.575808
CA	Barra de Guaratiba	22.993756/44.590284
CB	Barra de Guaratiba	22.994050/43.592963

*a*: BP1 - cattle burier site; *b*: coordinates obtained from Google Maps^®^. BP2: site of death of a contaminated bovine (24 h); BP3: grass covered soil; CA: corral A soil; CB: corral B soil; CT: Army Technology Center (CTEx) soil.

Soil samples (20 g) were collected with sterile stainless-steel spatulas at 2 to 5 cm depth. Sampling areas with 50 m^2^ were delimited and subsamples were collected at the angles (A, B, C and D) and at the central point (E), totalising 100 g in each collection site (Figure). These samples were stored at 4°C until processing. 

**Figure:**
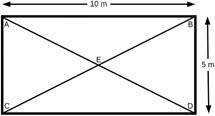
Soil sample collection scheme

*Physicochemical analysis of soil samples* - Physicochemical characterisation of soil samples was performed at the Analytical Centre Laboratory of the Federal Rural University of Rio de Janeiro (UFRRJ). The main features for *B. anthracis* spore persistence in soil, such as granulometry, Ca^2+^ concentration, pH and organic matter content were analysed. Values suggested by the Brazilian Agricultural Research Corporation (EMBRAPA) and the Commission of Soil Fertility of Minas Gerais State (CFSEMG) were used for comparison.^14^ ^,^ ^15^ The definition of soils textural class was based on the Triangle Diagram from the United States Department of Agriculture (USDA).^16^

*Soil processing -* All samples were submitted to heat treatment at 70ºC for 15 min in water bath. Serial dilutions were performed in SATAMP up to 10^-9^. From each serial dilution, 100 µL were transferred and plated with Digralski loops in the culture media and incubated at 33°C for 24 h. Culture media used in this study were: nutrient agar (NA) (Difco™), NA with 0.25 M sodium acetate (NA+) (Difco™), Columbia agar base (Difco™) with 5% of sheep blood (CBA), CBA with 0.25M sodium acetate (CBA+), CBA with 0.5% sodium bicarbonate (CBA++) and PLET agar (Sigma-Aldrich - 15 g/L agar, 30,000 U/L polymyxin b, 300,000 U/L lysozyme, 300 mg/L EDTA and 40 mg/L thallium acetate). Samples were homogenised with manual rotational motion for 30 min. Subsequently, 1 g of soil was transferred to 100 mL of sterile buffered saline (0,2 M potassium hydrogen phosphate - SATAMP - pH 7.2) and homogenised at 33°C for 30 min at 150 rpm (Shaker Innova 4080, New Brunswick Scientific Co., USA).

Colonies presenting characteristic *B. anthracis* morphology were selected and submitted to microscopic examination using Gram staining. Gram-positive bacteria presented suggestive characteristics of *B. anthracis* (“box-shapped” cells with 1-1.5 μm width and 4-10 μm length and subterminal, cylindrical and non-deforming spores) were further characterised as described below.

*Identification of isolates suspected of B. anthracis* - Isolates presenting *B anthracis* characteristic colonies and cell morphology were further evaluated for gamma phage and penicillin susceptibility, motility and presence of *pag* and *cap* genes by specific PCR and MALDI-TOF/MS (Microflex LT mass spectrometer, Bruker, USA) as described below.

*B. anthracis* (CCGB 1861) was used as positive control and *B. cereus* (CCGB 0406 LFB-Fiocruz 406) as negative control. Both strains were provided by Collection of Genus *Bacillus* and Related Genera-CCGB-Fiocruz.

The strains received a numeral code for identification (Table II). The results of the 52 isolates are shown in Table II.

**TABLE II:** Results of 52 isolates

Collect location	Culture media	Codes	Arrangement chains	Predominant sporangium	Predominant spore	Motility test	MALDI-TOF^*a*^	Susceptibility to penicillin	Susceptibility to gamma phage	Haemolysis	PCR pXO1	PCR pXO2
CT	CBA	8	Short	St, Nd, Ci	Ws	-	1.636 NI	R	S	A	-	-
		32	Long, isolated cells and in pairs	St, d, Ci	Ci	-	1.959 Ba	R	S	A	-	-
		40	Long	St, Nd, Ci	Ci	-	1.988 Ba	S	R	A	-	-
	CBA++	2	Short	Ws	Ci	-	1.949 Ba	R	S	A	-	-
	CBA+	3	Long	St, Nd, Ci	Ci	-	2.016 Ba	R	S	A	-	-
		5	Long and short	St, Nd, Ci	Ws	-	2.078 Ba	R	R	A	-	-
		10	Long, isolated cells and in pairs	St, Nd, Ci	Ws	-	1.424 NI	S	R	Beta	-	-
	NA	4	Long	St, Nd, Ci	Ci	-	2.065 Ba	R	S	A	-	-
		33	Short	St, Nd, Ci	Ws	-	1.948 Ba	S	R	Beta	-	-
		9	Long	St, Nd, Ci	Ci	-	2.000 Ba	R	R	A	-	-
CT	NA	41	Long	St, Nd, Ci	Ci	+	1.856 Ba	R	R	Beta	+	-
	NA+	20	Long	Ws	Ws	-	1.891 Ba	S	R	Beta	-	-
		21	Long	St, Nd, Ci	Ws	-	1.906 Ba	R	R	A	-	-
	PLET	22	Short	St, Nd, E	Ws	-	1.861 Ba	R	R	Alfa	-	-
		24	Short	St, Nd, Ci	Ws	-	1.922 Ba	S	R	A	-	-
		34	Short	St, Nd, Ci	Ci	-	1.608 NI	S	R	A	-	-
		36	Short	St, Nd, Ci	Ws	-	1.817 Ba	R	S	A	-	-
		37	Isolated cells and in pairs	St, Nd, Ci	Ws	-	1.672 NI	R	S	A	-	-
		31	Long in curve, isolated cells and in pairs	St, Nd, Ci	Ci	-	2.006 Ba	S	R	A	-	-
		39	Short	St, Nd, Ci	Ws	+	1.969 Ba	R.	R	Alfa	-	-
		42	Short	St, Nd, Ci	Ci	-	1.700 Ba	S	R	A	-	-
CB	NA+	18	Long	St, Nd, E	Ws	+	2.068 Ba	S	R	A	-	-
CA	NA	6	Long, isolated cells and in pairs	St, Nd, Ci	Ws	-	1.832 Ba	R	S	A	-	-
		11	Long	St, Nd, Ci	Ci	+	1.435 NI	S	R	A	-	-
		17	Long, isolated cells and in pair	St, Nd, Ci	Ci	-	1.761 Ba	S	R	A	-	-
		38	Long and short	St, Nd, Ci	Ws	-	2.045 Ba	S	R	A	-	-
	PLET	7	Short, isolated cells in pairs, suggesting poles with right angles	St, Nd, Ci	Ci	-	1.941 Ba	R	S	A	-	-
		23	Long	St, Nd, Ci	Ci	-	1.365 NI	R	R	Alfa-	-	-
		25	Curved shorts, isolated cells and in pairs	St, Nd, Ci	Ws	-	1.903 Ba	S	R	A	-	-
		26	Curved shorts, isolated cells and in pair	St, Nd, Ci	Ci	-	1.729 Bp	S	R	A	-	-
		28	Shorts, isolated cells and in pair	St, Ld, Ci	Ci	-	1.988 Ba	S	R	A	-	-
		29	Short, isolated cells in pairs, suggesting a right-angle pole	St, Nd, Ci	Ci	-	2.315 Ba	S	S	A	+	-
		30	Short, isolated cells in pairs, suggesting a right-angle pole	St, Nd, Ci	Ws	-	1.944 Ba	R	S	A	-	-
BP1	CBA	51	Long	St, Nd, Ci	Ci	-	2.043 Bm	S	R	A	-	-
	CBA+	55	Long	St, Nd, Ci	E	-	1.73 Ba	S	R	Beta	-	-
		66	Long, isolated cells in pairs, suggesting poles with right angles	St, Nd, Ci	Ci	+	1.518 NI	R	R	A	-	-
		84	Long, isolated cells in pairs	St, Nd, Ci	Ci	-	1.634 NI	R	R	A	-	-
	PLET	69	Short	St, Nd, Ci	Ws	-	1.417 NI	R	R	A	-	-
		72	Short	St, Nd, Ci	Ws	+	2.022 Ba	R	R	Beta	-	-
		73	Short, cells in pairs	St, Nd, E	Ws	-	1.563 NI	R	R	A	-	-
		81	Short, isolated cells and in pairs	St, Nd, Ci	E	+	2.212 Bl	R	R	A	-	-
		82	Short, isolated cells and in pairs	St, Nd, Ci	Ci	-	1.619 NI	R	R	A	-	-
		90	Short, isolated cells and in pairs	St, Nd, E	Ws	+	1.308 NI	R	R	A	-	-
		93	Short, isolated cells and in pairs	St, Nd, Ci	Ws	+	1.204 NI	R	R	Beta	-	-
		97	Short, isolated cells	St, Nd, Ci	Ci	+	1.428 NI	S	R	A	-	-
BP2	PLET	49	Short	St, Nd, Ci	Ci	-	2.073 Ba	R	R	Beta	-	-
		53	Short, isolated cells and in pairs	St, Nd, Ci	E	-	1.853 Ba	R	R	Beta	-	-
		59	Short	St, Nd, Ci	E	+	1.335 NI	R	R	Beta	-	-
		78	Short, cells in pairs	St, Nd, Ci	Ci	+	2.053 Ba	R	R	Beta	-	-
BP3	CBA+	52	Long and short	St, Nd, Ci	Ci	+	1.624 NI	R	R	Beta	-	-
	PLET	85	Short, isolated cells and in pairs	St, Nd, Ci	Ws	+	1.591 NI	S	R	A	-	-
		95	Short, isolated cells	St, Nd, Ci	Ci	-	1.853 Pt	R	R	A	-	-
Vaccine	CBA	PC	Short, isolated cells suggesting a right-angle pole	St, Nd, Ci	Ci	-	2.4 Ba	S	S	A	+	-
BC	CBA	NC	Long	C, Nd, Ci	Ci	+	2.301 BC	R	R	Beta	-	-

*a*: higher score obtained when comparing the mass spectrum of the sample with the microbial species presented. A: absence; Ba: *Bacillus anthracis*; Bc: *B. cereus*; Bl: *B. licheniformis*; Bm: *B. marisflavi*; BP1: sample from where bovine was buried; BP2: sample from the place where the bovine was left after death (24 h); BP3: grass sample; Bs: *B simplex*; C: central; CA: (corral A’ soil ; CB: (corral B’ soil); CBA: Columbia agar base added 5% defibrinated ram blood; CBA+: Columbia agar base added 5% defibrinated sheep blood (0.25 M sodium acetate); CBA++: Columbia agar base added % sheep defibrinated blood with 0.5% sodium bicarbonate; Ci: cylindrical; CT: Army Technology Center (CTEx) soil; E: elliptical; Long: have more than six cells; NA: nutrient agar medium without containing NaCl; NA+: nutrient agar medium without containing NaCl and 0.25 M sodium acetate; NC: negative control; Nd: no deformans; PC: positive control; PLET: agar (Sigma-Aldrich - 15 g/L agar, 30,000 U/L polymyxin b, 300,000 U/L lysozyme, 300 mg/L EDTA and 40 mg/L thallium acetate); Pt: *Paenibacillus thiaminolyticus*; R: resistant; S: susceptibility; SD: slightly deforming; Se: without sporangia; Short: have up to sex cells; St: subterminal; Ws: without spore.

*Motility test -* To evaluate motility a 1 µL bacterial loop of each isolate was inoculated in 5 ml of nutrient broth with 0.5% of D-(+)-glucose and incubated in anaerobic conditions at 33°C for 20 h. Motility was observed on a fresh exam of broth culture by optical microscopy (x1000) (NiKon, Japan).

*Susceptibility to gamma phage and penicillin -* For the gamma phage susceptibility assay, 1 µL of 7.9 × 10^9^ PFU/mL gamma phage suspension [provided by Public Health Agency (HPA), Porton Down, England] was used. For the penicillin susceptibility test, 10 U.I. Sensibiodisc (CECON, Brazil) were used. Samples grown in CBA were inoculated in nutrient broth and incubated at 33ºC for 24 h. Bacterial suspensions were inoculated into CBA plates using sterile swab. After the inoculation, penicillin disks and a 10-µL drop of gamma phage suspension were placed on the surface and plates were incubated at 37ºC for 24 h in aerobic conditions. Absence of bacterial growth as lysis plates and inhibition zones around the penicillin disk of any size were considered as positive results.

*Microbial identification by MALDI-TOF/MS -* Mass spectra were acquired using a Microflex LT mass spectrometer and results were analysed using Biotyper software version 3.1 (Bruker Daltonics, USA) containing a bioterrorism biological agent library, including 23 virulent *B. anthracis* strains and the Sterne strain.

Sample inactivation was performed according to Lasch et al.^17^ Briefly, 1 µL of each sample was transferred to a 96-well stainless-steel plate and allowed to dry at room temperature. Then, 1 µL of α-cyano-4-hydroxycinnamic acid matrix (HCCA) was added and allowed to dry at room temperature. MALDI-TOF MS analysis was performed in duplicate by linear mode with the following parameters: laser frequency 20 Hz; voltage of ion sources 1 and 2, 20 kV and 18.6 kV, respectively; molecular weight range 2,000 to 20,000 Da. Mass spectra were collected through the sum of 240 laser shots, acquired through four groups of 60 shots cast in randomly selected distinct regions. The spectra of the samples were calibrated internally, using *Escherichia coli* DH5α (IDQBRN, Brazil) ribosomal proteins as reference. Spectra were processed by Biotyper software in standard operating mode, which performs the research in its reference library in order to get the best match for the sample. Results were scored ranging from 0 to 3.0. Scores below 1.7 were considered unreliable, those between 1.7 and 2.0 were considered possible indicator of genus, those between 2.0 and 2.3 were considered reliable for genus and possible for species identification and those above 2.3 were considered reliable for identification at the species level.

*Evaluation of pag and cap genes in isolates suspected of B. anthracis -* To survey the presence of *pag* and *cap* genes, a multiplex PCR was performed using *Beyer 8* (TCC-TAA-CAC-TAA-CGA-AGT-CG), *Beyer 5* (GAG-GTA-GAA-GGA-TAT-ACG-GT), *1234* (CTG-AGC-CAT-TAA-TCG-ATA-TG*)* and *1301* (TCC-CAC-TTA-CGT-AAT-CTG-AG) primers.^3^ ^,^ ^18^

DNA extraction was carried out using Instagene matrix kit (Bio-Rad, USA) according to the manufacturer’s instructions. PCR reactions were performed as previously described.^17^ ^,^ ^18^ Briefly, multiplex PCR reactions for *pag* and *cap* were performed in 25 µL reaction volume containing 2.5 µL of template DNA, 2.5 µL of 10 × PCR Buffer, 0.5 µL of DNTP, 1,25 µL of a 10 µM solution of each primer, 14.38 µL of DNAse free water (Thermo Fisher Scientific, USA) and 0,0125 µL of Taq. For amplification, the following parameters were used: an initial denaturation step of 95°C for 5 min; 30 cycles of 95°C for 1 min; 55°C for 30 sec; 72°C for 30 sec; and a final extension step at 72°C for 5 min.^19^ ^,^ ^20^

PCR products were observed after 1% agarose gel electrophoresis containing ethidium bromide under ultraviolet light.

*PCR amplicon sequencing -* Isolates positive for *pag* genes were submitted to simplex PCR, as described above, with the *pag* primers. PCR products were purified using Exosap-IT Express (Thermo Fisher Scientific, USA), according to the manufacture instructions. Amplicons were sequenced in an ABI3730XL automated sequencer (Applied Biosystems). Forward and reverse sequences were aligned and edited using Seqman program (DNAstar, Larsegene, version 7.0) and the data compared to the sequences deposited in GenBank with nBLAST. 

*16S rRNA gene sequencing -* Isolates positive for *pag* genes and those susceptible to gamma phage were submitted to amplification and sequencing of the gene encoding the 16S rRNA, according to Watts et al.,^21^ with universal primers pA (5′-AGA-GTT-TGA-TCC-TGG-CTC-AG), pH (5′-AAG-GAG-GTG-ATC-CAG-CCG-CA), *1831* (5′-GAG-GAA-CAC-CGA-TGG-CGA-AGG-C) and *1832* (5′-GCC-CCC-GTC-AAT-TCC-TTT-GAG-TT). 

Products were purified using Exosap-IT Express (Thermo Fisher Scientific, USA), according to the manufacture instructions and sequenced at the DNA Capillary sequencing Facility - SANGER (Fiocruz, Brazil) in an ABI3730XL automated sequencer (Applied Biosystems). Forward and reverse sequences were aligned and edited using BioEdit 7.2 software and data were compared to the sequences in GenBank using nBLAST. The sequences were submitted at GenBank.

## RESULTS

*Physicochemical characteristics of soil samples -* The textural class of all samples was identified after particle size analysis. Samples BP1 and BP2 were classified as Loam: BP3 as Sandy Clay Loam, CA as Clay loam; CB and CT as Sandy Loam (Table III).

**TABLE III:** Physicochemical characteristics of soil samples

Properties	BP1^*a*^	BP2^*a*^	BP3^*a*^	CA^*a*^	CB^*a*^	CT^*a*^
Granulometry g/kg	Sand 459 g, Silt 297 g, Clay 244 g	Sand 506 g, Silt 301 g, Clay 193 g	Sand 470 g, Silt 229 g, Clay 301g	Sand 390 g, Silt 235 g, Clay 375 g	Sand 637 g, Silt 196 g, Clay 167g	Sand 778 g, Silt 156 g, Clay 66 g
pH	6,8 weak acidity	5,2 average acidity	5,1 average acidity	5,7 average acidity	5,8 average acidity	5,9 average acidity
Ca^2+^ cmol/dm^3^	6,7 high content	0,7 low content	0,3 low content	4,7 high content	8,8 high content	2,8 average content
Organic matter g/dm^3^	68,3 high content	25,2 average content	25,0 average content	25,5 average content	30,3 high content	34,3 high content
Textural class	Loam	Loam	Sandy clay loam	Clay loam	Sandy loam	Loamy sand

*a*: BP1 - cattle burier site. BP2: site where the bovine was left after death for 24 h; BP3: grass sample; CA: corral A soil sample; CB: corral B soil sample; CT: *Army Technology Center* (CTEx) soil sample.

Regarding pH analysis, BP1 presented weak acidity while the other samples presented medium acidity.

Results for Ca^2+^ analysis showed that, according to the parameters suggested by EMBRAPA,^15^ BP1, CA and CB presented high Ca^2^+ content (> 3 cmol/dm3), CT, medium Ca^2+^ content (1-3 cmol/dm3) and BP2 and BP3 presented low Ca^2+^ content (< 1 cmol/dm3).

Regarding organic matter content, samples BP1, CB and CT presented high levels (> 3.0 dag/kg or > 30.0 g/dm3), while, BP2, BP3 and CA presented average levels (1.5-3.0 dag/kg or 15-30 g/dm3), according to EMBRAPA.^15^

*Isolation and identification of B. anthracis suspected bacteria -* Bacterial growth was observed after incubation in aerobiosis. After observation of macroscopic characteristics, 369 colonies were Gram stained and 52 isolates were selected based on morphological and staining characteristics, as shown in Table IV.

**TABLE IV:** Number of colonies selected according to macroscopic and microscopic characteristics and in each culture media

Samples	Culture medium												Total	
	NA		NA+		CBA		CBA+		CBA++		PLET				
	Mac	Mic	Mac	Mic	Mac	Mic	Mac	Mic	Mac	Mic	Mac	Mic	Mac	Mic
BP1 soil	12	0	10	0	12	1	9	3	9	0	9	8	61	12
BP2 soil	13	0	12	0	10	0	8	0	9	0	4	4	56	4
BP3 soil	10	0	10	0	12	0	9	1	8	0	10	2	59	3
CT soil	14	4	10	2	13	3	11	3	10	1	12	8	70	21
CA soil	13	4	12	0	10	0	12	0	10	0	10	7	67	11
CB soil	11	0	9	1	13	0	10	0	9	0	4	0	56	1
Total	73	8	63	3	70	4	59	7	55	1	49	29	369	52

NA: nutrient agar without NaCl; NA+: nutrient agar with 0.25 M sodium acetate; CBA: Columbia agar base added 5% defibrinated sheep blood; CBA+: Columbia agar base added 5% defibrinated sheep blood with 0.25 M sodium acetate; CBA++: Columbia base agar added 5% sheep defibrinated blood with 0.5% sodium bicarbonate; PLET: agar polymyxin B, lysozyme, EDTA and thallium acetate; Mac: macroscopic; Mic: microscopic.

*Phenotypic and genomic identification of B. anthracis suspect isolates -* From 52 isolates, 16 were negative for motility test, one strain showed susceptibility for gamma phage and penicillin and 10 were gamma phage-susceptible and penicillin-resistant. MADI-TOF/MS identified one isolate as *B. anthracis* (score above 2.3) and 11 isolates as belonging to the genus *Bacillus*. Two strains, 29 and 41, showed amplification of *pag* gene, *pag* amplicons from isolates 29 and 41 showed 100% and 99.6% identity to pXO1, respectively.

Isolate 29 was identified as*B. anthracis*(not motile, PCR positive for *pag*, non-haemolytic, gamma phage/penicillin-susceptible and MALDI-TOF score 2.315) and isolate 41 was not identified as *B. anthracis,* but was placed in *B. cereus* group (motile, PCR positive for *pag*, haemolytic, gamma phage and penicillin-resistant and MALDI-F score 1.849) and was considered as *B. anthracis-*like bacterium.16S rRNA gene sequencing identified isolate 29 as belonging to *B. cereus* group showing 100% of identity and query cover with *B. anthracis* ATCC141576 (GenBank accession MT994366), while isolate 41 presented 100% identity and 94% query cover with eight species belonging to the *B. cereus* group (GenBank accession MT994363 - *Bacillus cereus* strain IAM 12605, *B. pacificus* strain MCCC 1A06182, *B. paranthracis* strain MCCC 1A00395, *B. cereus* strain CCM 2010*, B. cereus* strain NBRC 15305, *B. cereus* ATCC 14579 (16S RNA mA), *Bacillus cereus* ATCC 14579 and *B. cereus* strain JCM 2152). In addition, isolates 2, 3, 4, 6, 7, 8, 30, 36 and 37 (all gamma phage-susceptible and penicillin resistant, pXO1- and pXO2-) were identified as belonging to the *B. cereus* group (GenBank accessions: MT994549, MT993863, MT994256, MT993936, MT993895, MT994361, MT994451, MT994163 and MT993931, respectively) (Table II and Table V).

**TABLE V:** Results of phenotypic and molecular identification tests of 52 isolates

Tests of isolates		Number of isolates in each test
Motility test	Non-motile	36
	Motile	16
MALDI-TOF	Score above 2.3	1 identified as *Bacillus anthracis*
	Score values between 2.0-2.3	11 confirmed as *Bacillus* sp.
	Score values between 1.7-2.0	22 identified as probable *Bacillus* sp.
	Score values lower than 1.7	18 were not identified
Susceptibility to gamma phage and penicillin	Susceptible to gamma phage and penicillin	1
	Susceptible to gamma phage and resistant to penicillin	10
	Resistant to gamma phage and penicillin	13
	Resistant to gamma phage and susceptible to penicillin	28
PCR	pXO1+/pXO2-	02
	pXO1-/pXO2-	50
Amplicon sequencing	Identity to *pag* (surogate of pXO1)	Isolate 29 (100% identity)
		Isolate 41 (99.6% identity)
16sRNA	Identified as strain belonging to *B. cereus* group	Isolate 29 (100% identity and 100% query cover to *B. paramycoides* strain MCCC 1A04098, *B. albus* strain MCCC 1A02146 and *B. anthracis* ATCC141576
		Isolate 41 (100% identity and 94% query cover to eight species belonging to the *B. cereus* group)
		Isolates 2, 3, 4, 6, 7, 8, 30, 36 and 37 (match to several species belonging to the *B. cereu*s group)

## DISCUSSION

In this study, one *B. anthracis strain* (harbouring pXO1, but not pXO2), one strain of *B. cereus* group harboured a pXO1-like plasmid (99.6% similarity with *pag*) and 10 *Bacillus* strains were isolated (Table V) from different soils of Brazil. These 10 isolates might be *B. anthracis* strains lacking virulence plasmids, which is in agreement with the findings of Kolton et al.^22^ The probability of positive results for *B. anthracis* species cannot be ruled out, as there are reports of such occurrence in the literature.^23^ ^,^ ^24^ These isolates were recovered from six different culture media. From PLET cultures, a pXO1+ *B. anthracis* was isolated, from NA, a pXO1+ *B. anthracis-like* was isolated. These data corroborated what is described in literature, which suggest PLET is the most selective medium for *B. anthracis* isolation.^6^

This study is the first on this subject performed in Brazil. It showed the importance of active surveillance in soil and the correct identification of the isolates for better understanding *B. anthracis* distribution in nature and for the elucidation of possible outbreaks in Brazil.

Several methods for isolation and identification *B. anthracis* in soil have been reported, although there is no consensus among the respective studies. A direct method was chosen, because it is a simple and economical protocol for survey *B. anthracis* in soil samples. Among the soils analysed in this study, the sample presenting characteristics that would favour the prevalence of *B. anthracis* spores was BP1, in according to Shadomy et al.^25^ Interestingly, neither *B. anthracis* nor *B. anthracis*-like strains were isolated from this sample. In the USA, the bacterium was isolated from soils with neutral to alkaline pH and high concentrations of nitrogen, while in Germany, there was no correlation with the type of soil. These differences indicate that the occurrence depends not only on the type of soil, but also on environmental conditions, the pathogen’s life cycle, persistence, ecology and the ability to lose or acquire virulence attributes.^6^ ^,^ ^26^ *B. anthracis* (harbouring pXO1, but not pXO2, strain 29) was isolated from the CA soil, a clay loam, with medium acidity, high Ca^2+^ and medium organic matter content. *B. anthracis* was isolated in the former USSR from soils with medium acidity.^26^ An isolate (strain 41) from CT soil cultured in NA presented microscopic characteristics of large *Bacillus* was resistant to penicillin and gamma phage, motile and beta haemolytic and would not be regarded as *B. anthracis*. However, this isolate harboured a pXO1-like plasmid (99.6% similarity with *pag*) and might be a *B. anthracis*-like bacterium. It was described that 7% of 1,000 *B. cereus sensu lato* isolated from environmental samples (soil, water, insects, plants) were shown to contain pXO1-like and/or pXO2-like plasmids. *B. mycoides* harbouring pXO1-like and/or pXO2-like plasmids is less related than *B. cereus* or *B. thuringiensis*. It is relevant to note that *B. anthracis*-like strains have already been described causing disease with the same symptoms as anthrax in animals and humans, including being lethal. This suggested the occurrence of plasmid transfer events across the different species of the *B. cereus* group.^27^

Species allocated in the *B. cereus* group have high genetic identity, which makes the differentiation complex, even by means of molecular tests.^28^ ^,^ ^29^ In this group of bacteria, species classification is still based on phenotypic and genotypic traits, pathogenicity, host preference and ecological niche.^23^ Phenotypic tests are essential for *B. anthracis* identification. However, those considered to be classic phenotypic characteristics may not be present in all *B. anthracis* strains. Therefore, Kovac et al.^30^ proposed phylogenetic classification based on the complete genome sequencing for bacteria belonging to the *B. cereus* group. The isolation of 10 strains presenting all the phenotypic characteristics of the specie, but not harbouring pXO1 neither pXO2 corroborated what were described in the literature.

Molecular assay in MALDI-TOF MS analysis, the only strain with a score above 2.3, which confirms species identification, was the one isolated from CA soil (strain 29). This isolate was confirmed as *B. anthracis* by other methods applied in this study. Among the isolates analysed, several were identified only at genus level, while others were identified as non-*Bacillus*, although the results of physiological tests had identified them as *Bacillus* spp.

Due to the inherent difficulty in differentiating between species with such a genomic similarity, it is necessary to enlarge the number of spectra in MALDI-TOF library in order to achieve better results for *B. anthracis* and *B. anthracis*-like bacteria identification.^30^ Although PCR has the potential to infer the presence pXO1 and pXO2 plasmids, the method does not inform the species and the lineage unequivocally. Total genome sequencing has been shown to be the best methodology to study geographical distribution of strains during natural outbreaks or events suspected of bioterrorism.^22^ ^,^ ^24^ 16sRNA sequencing showed that it is difficult to differentiate strains that belong to the *B. cereus* group. These results are due to the remarkable 99.6% identity percentage what makes them indistinguishable from each other when 16sRNA gene subunit sequencing was made.^23^ ^,^ ^24^ ^,^ ^29^ These results showed the importance of whole genome sequencing for identification of bacteria belonging to the *B. cereus* group.

To date, the literature describes that virulent strains of *B. anthracis* necessarily harbour both plasmids. However, strains of *B. cereus* harbouring plasmid-like may cause fatal inhalation infection similar to inhalation anthrax, and cases in humans have already been described. Due to Brazil’s continental dimensions, a large diversity of soils and climates can be found. The samples analysed represent only a fraction of this diversity. Nevertheless, it is important to highlight that this work showed promising results and it was the first study to report results from an active surveillance for *B. anthracis* in Brazil.

In conclusion, since livestock is one of the main economic activities in Brazil, large-scale studies involving *B. anthracis* active surveillance in soil in Brazil should be performed to reduce and/or prevent economic losses, and a complete characterisation of the isolates should be carried out in order to provide accurate information about Brazilian strains. In this work, we isolated *B*. *anthracis* (pXO1^+^ and pXO2-) and *B. anthracis*-like in soil samples, being the first description of this type in Brazil. This data increases the need to carry out surveillance of these species in the soil. The correct identification of these species is of paramount importance for the knowledge of the pathogen distribution in nature and elucidation of possible outbreaks.
